# Supplementary material for: Chromothripsis during telomere crisis is independent of NHEJ, and consistent with a replicative origin
Source: Genome Res. 2019 May;29(5):737–49. doi: 10.1101/gr.240705.118 (PMC6499312; doi:10.1101/gr.240705.118)
Supplement: Supplemental Material [file supp_gr.240705.118_Supplemental_file_1.zip › contigs/annotated_contigs/DB93/contig.6.DB93_length_1077_mean_cov_21.2451253482.docx]

**DB93_length_1077_mean_cov_21.2451253482**

```
GCTTACAAAAATGCATGCAGTATAATAAAAGAGAAAA|AAAAAGGT|TTACACAAAAAGAGACAGAAAGATCGAGGGCGCAAACGAGAA
 >chr9:79453072-79453117 - E=4e-15 p=2e-02 >chr7_gl000195_random:56997-57368 - E=7e-201
AAACCCAGTAGGAGAAAAATTAGAGCTTTAAAAAAATCTTGACAGGGTGCGGTGGCTCACACCTGTAATCCCAGCACTTTGGGATGCTG

AGACGTGTGGATCGCCTGAGGTCAAGGGTTTGAAACCAACCTGGACAACATGGTGAAACCACCGTCTCTTCTAAAAAATACAAAATGAG

CCAGATGTGGTGGCGCATGCCTGTAATCCCAGCTACTTGGGAGACTGAGGCAGGAGAATCAATTGAACCTGGGAGGCAAAGGTTCCAAT

AAGCCGAGATCACACCACTGCACTCCTGCCTGGGATACAAGAGTGAAACTGTGTC|AAAAAAATTG|ACATCCCCTCTATTTAGAGAAC
                                                                  >chr17:50489790-50489
GCCTAATCATCTATAGCTGGGTCCCCAAAGATTTCTAAACTTTCTA|TTTTCAG|CACTTTGAGTATGTCATCCTACAACCTCTTACCT
865 - E=4e-33                                  >chr7_gl000195_random:67667-67765 - E=6e
GTAAGTTTTTTTGCTGAGACATCTGCTGGTCATCCTGCAGGGGTAAACTTGTACATG|TATAT|TTTTATGTACAGTATATAATATATA
-47                                                            >chr8:43096056-43096158
GTTTGTGAACTTTGATATTTCATGTAGAGTATATAATGTATATTTGGGCTTCTTTGGTATTTAATGTACAGTATATA|A|TTGTCGGTG
- E=3e-16                                                                      >chr7_gl
GGCTTCAGAACTATAATCACAGCATGTACCGGGACCCGGGGTGCATAGCCCTGGAGGGAGGGGCAGAGCGGTGTGGACTTCCCGGCGTC
000195_random:30147-30531 - E=7e-207
CAAAGCACTGCTGAGTTCTTATGCTATGCCGACTCTTTGCCAAGGAGAATGAGTACATAGGTGGACCTAGAGGACAGGTCTGCGCTGAG

CATTGAGGAGGGTATTACCCTACATAGGCACCTTACCTTTGCCCAAATCGGGCGGGTTGTCCTCAACTGATTGGCCCTATCCTTCTCAA

GTTCCTCTTTCAGCTGCACCCAGGGTTCTTTCCAGAGCATTGCGCCTTCTGCAGCACAGGGCGCTGCCTTCTTTCCTAAACTGCTGTGG

AAACTGTCCTGATGTCTGA
```
